# Supplementary material for: Prolonged Hyperoxygenation Treatment Improves Vein Graft Patency and Decreases Macrophage Content in Atherosclerotic Lesions in ApoE3*Leiden Mice
Source: Cells. 2020 Feb 1;9(2):336. doi: 10.3390/cells9020336 (PMC7072413; doi:10.3390/cells9020336)
Supplement: Supplementary file 1 [file cells-09-00336-s001.pdf]

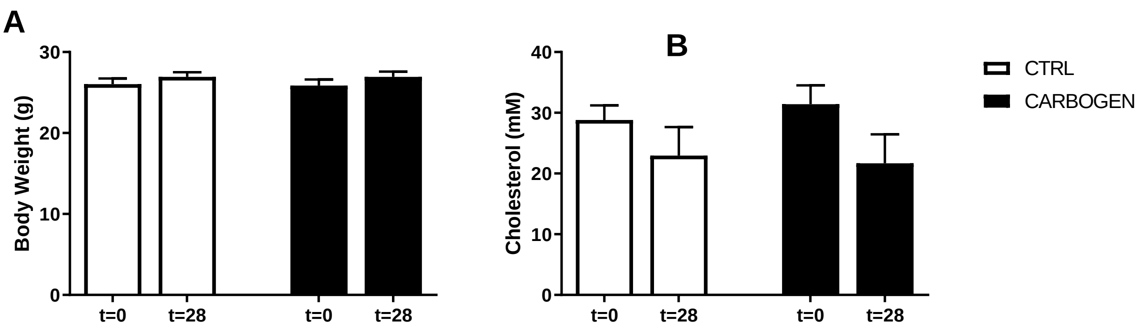

Figure S1. Bodyweight and cholesterol levels acute carbogen treatment. (A). Bodyweight before (t=0) and 28 days after surgery (t=28) of control and one-time carbogen treated mice. (B). Plasma cholesterol levels before (t=0) and 28 days after surgery (t=28) of control and one-time carbogen treated mice.

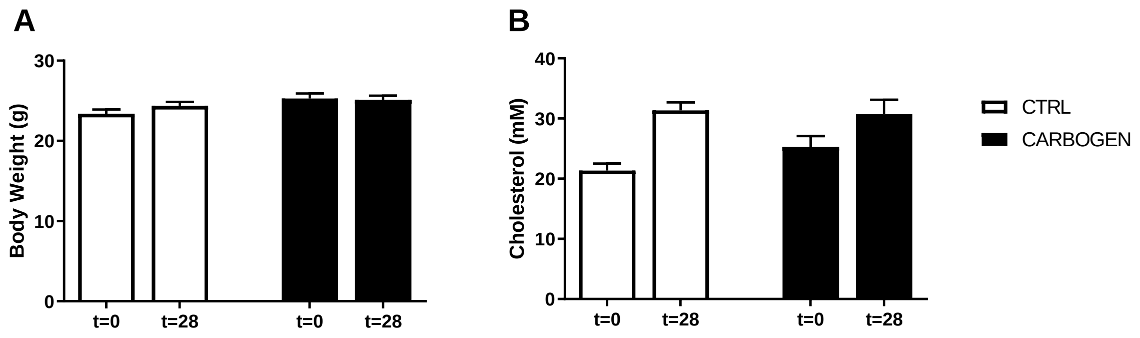

Figure S2. Bodyweight and cholesterol levels chronic carbogen treatment. (A). Bodyweight before (t=0) and 28 days after surgery (t=28) of control and prolonged carbogen treated mice. (B). Plasma cholesterol levels before (t=0) and 28 days after surgery (t=28) of control and prolonged carbogen treated mice.

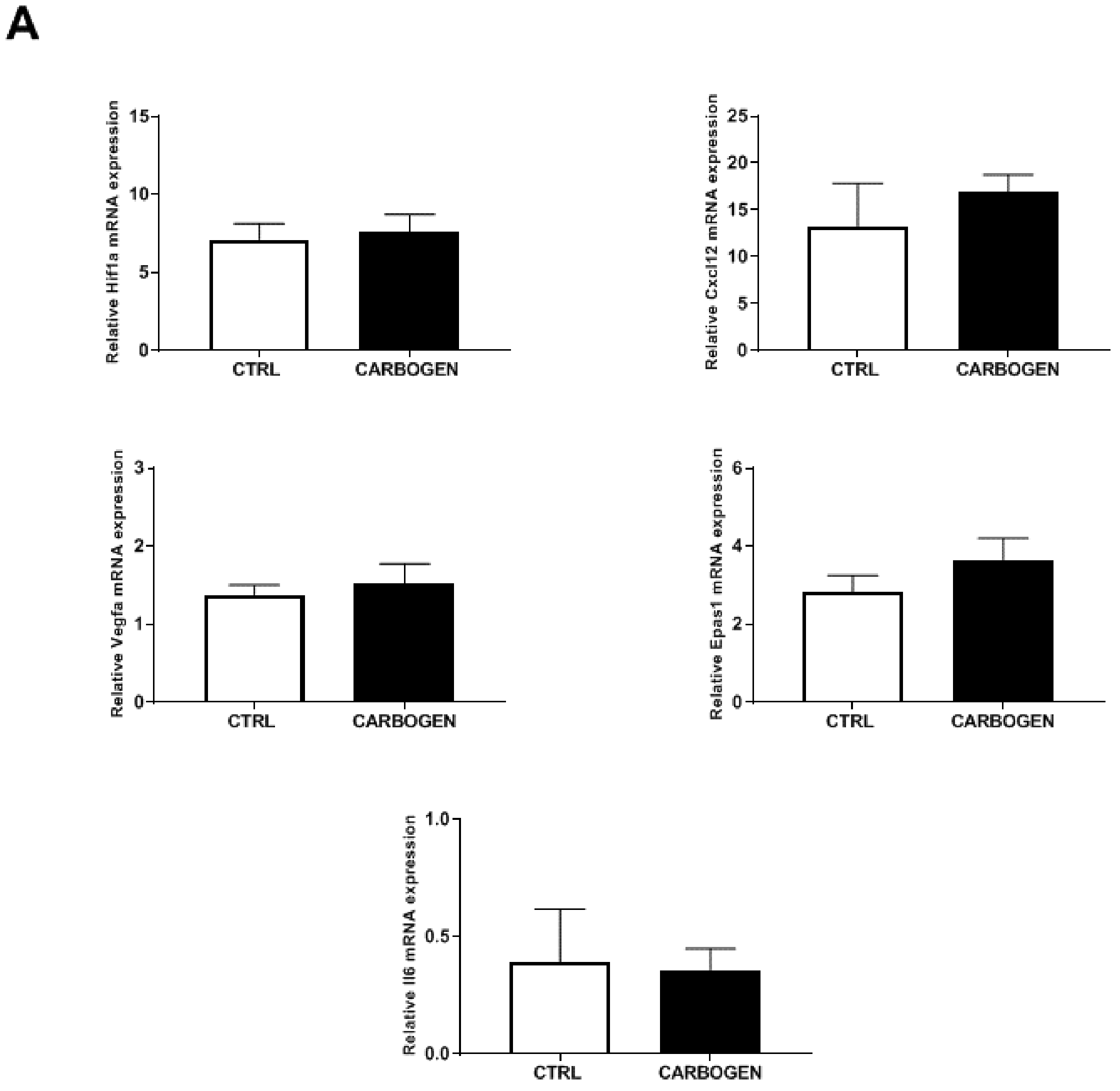

Supplemental figure 3. Total wall gene expression. (A) Expression of Hif1a, Cxcl12, Vegfa, Epas1 and Il6 in the mice from the control and one-time carbogen treated groups.

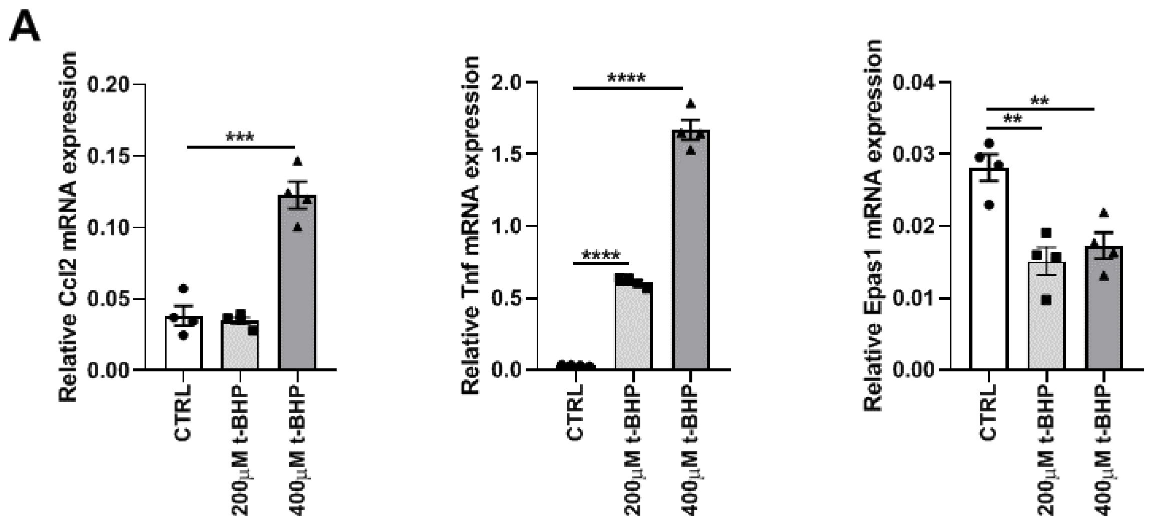

Supplemental figure 4. BMM gene expression. (A) mRNA expression of Ccl2, Tnf and Epas1 in control BMM and BMM treated with the ROS mimic t-BHP.

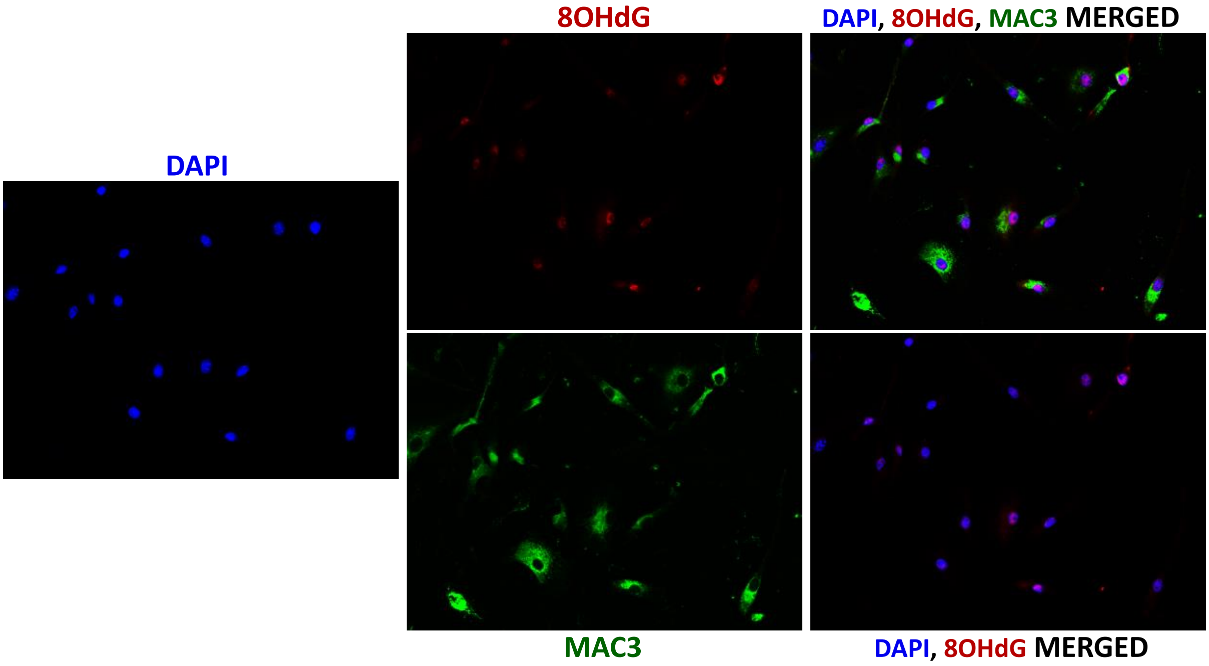

Supplemental figure 5. BMM 8OHdG IHC staining. Representative pictures of BMM treated with t-BHP are shown. Examples images stained for DAPI (blue), Mac3 (green) and 8OHdG (red) as well as a merged image are shown.
